# The effects of community education on tobacco use among older adults in China

**DOI:** 10.18332/tid/204512

**Published:** 2025-05-29

**Authors:** Shuang Yu, Yu Liu, Manyi Wang, Yinhe Liang

**Affiliations:** 1 Research Institute of Social Development, Southwestern University of Finance and Economics, Chengdu, China; 2 Institute of Chinese Financial Studies, Southwestern University of Finance and Economics, Chengdu, China; 3 Institute of Chinese Financial Studies, Tsinghua University, Beijing, China; 4 School of Economics and Management, Central University of Finance and Economics, Beijing, China; 5 Institute for Global Health and Development, Peking University, Beijing, China

**Keywords:** tobacco use, older adults, community education, instrumental-variable

## Abstract

**INTRODUCTION:**

Smoking among older adults is increasingly linked to chronic diseases and higher mortality rates. However, the influence of community education on smoking behavior in older adults remains understudied. This research examines the causal effect of community education on smoking habits of older adults in China.

**METHODS:**

We use four waves of longitudinal secondary data (2011, 2013, 2015, and 2018) from the China Health and Retirement Longitudinal Study (CHARLS), covering adult children aged 22–58 years and their parents aged ≥60 years. A two-stage least squares (2SLS) model is applied to estimate the causal impact of community education on smoking behaviors among older adults, using exposure to the 1986 Compulsory Schooling Law as an instrumental variable. Mechanism and subgroup analyses are further conducted to validate and interpret the estimated effects.

**RESULTS:**

This study includes 26489 adults aged ≥60 years. Community education significantly reduces the likelihood of ever smoking (β= -0.030; 95% CI: -0.048 – -0.012), current smoking (β= -0.020; 95% CI: -0.038 – -0.003), and the number of cigarettes (β= -0.038; 95% CI: -0.075 – -0.001). Mediation analysis indicates that these effects are partially explained by intergenerational support, well-being, and mental health. Among ever smokers, the Center for Epidemiologic Studies Depression Scale (CES-D) score shows the largest indirect effect (β=0.0004; 95% CI: 0.0000–0.0010), accounting for 3.5% of the total effect, followed by contact with children (β= -0.0003; 95% CI: -0.0010 – -0.0000; 1.8%) and optimism (β= -0.0001; 95% CI: -0.0002 – -0.0000; 0.7%). For smoking intensity, CES-D remains the only significant mediator (β=0.0008; 95% CI: -0.0003–0.0010), explaining 4% of the total effect.

**CONCLUSIONS:**

Community education plays a crucial role in lowering smoking rates among older adults. Policymakers should prioritize educational programs and enhance healthcare services to reduce smoking and improve public health outcomes for aging populations.

## INTRODUCTION

In recent years, smoking among older adults has attracted growing attention from both the government and academia due to its association with chronic conditions, including cardiovascular disease, chronic obstructive pulmonary disease, and various cancers^1^. Smoking contributes significantly to the global burden of non-communicable diseases, placing substantial strain on healthcare systems^2^. Furthermore, it remains a leading cause of death among older individuals, with over 70% of smoking-related deaths occurring among those aged ≥60 years^3^.

Previous studies have primarily focused on individual socioeconomic factors as determinants of smoking behavior in older adults^4-6^. For instance, Ma^7^ highlighted that adult children’s educational attainment can have intergenerational spillover effects on parents’ health, cognitive function, and smoking behavior. Some research also emphasizes the role of social characteristics, such as family structure and peer influence, in shaping smoking behavior^8,9^. However, limited attention has been given to the potential spillover effects of community education on smoking among older adults. This study addresses this gap by investigating the causal relationship between average level of education of adult children of other elderly people in the community (hereafter referred to as ‘community education’) and tobacco use in older populations.

Community education may become a key influence in older adults’ cognitive functioning. As the central domain of older adults’ daily lives, the community environment is closely linked to intergenerational interactions. Despite Confucian filial piety culture continuously emphasizing the ethical responsibility of intergenerational support, the family structure has changed in the context of the one-child policy, and the reduction in family caregiving resources has highlighted the role of community neighborhoods. Specifically, community education may affect smoking behavior through several theoretical channels. On the one hand, according to social capital theory, the education of social network members may influence the behavior of others. Interactions among older adults within the community can transmit health literacy and knowledge about the negative effects of smoking, thereby indirectly reducing smoking behaviors. On the other hand, according to neighborhood effect theory, the social and educational environment shapes individual behaviors and norms^10,11^. In communities where educational resources foster anti-smoking attitudes, these norms may create social pressure for all residents, including older adults, to reduce smoking^12^. Therefore, the educational environment of community children may influence smoking behaviors among older adults.

Our research seeks to address three key questions: 1) ‘To what extent does community education causally influence smoking behavior among older adults?’; 2) ‘What mechanisms mediate the relationship between community education and smoking behavior?’; and 3) ‘Does community education exert a moderating effect on tobacco use within older populations?’.

## METHODS

### Data

This study uses secondary data from the China Health and Retirement Longitudinal Study (CHARLS), conducted by the National School of Development at Peking University. A substantial body of research, including studies by Liu et al.^6^, Wang et al.^13^, and Chi et al.^14^, has utilized CHARLS to investigate the health status and smoking behaviors of older adults, demonstrating its reliability in geriatric public health research. CHARLS is a nationally representative survey targeting middle-aged and older populations in China (aged ≥45 years), providing detailed information on education, cognitive abilities, health, income, and other demographic characteristics. CHARLS uses a multi-stage stratified probability sampling method to ensure national representativeness. Based on 2009 census data and PPS (probability proportional to size) sampling, 150 counties and 450 villages/communities were selected across 30 provinces. All sampling was conducted in STATA, and no replacements were allowed.

The baseline survey was conducted in 2011, with follow-up waves in 2013, 2015, and 2018, resulting in four waves of data collected to date. By 2018, the survey included approximately 19000 respondents from 12400 households. To ensure data reliability and alignment with our research focus, we restricted the sample to communities with at least ten households, included only respondents aged ≥60 years, and excluded observations with missing control variables. After applying these criteria, the final sample sizes used in our study were 7208 in 2011, 8204 in 2013, 6285 in 2015, and 4792 in 2018.

### Sample selection criteria

This analysis combines data from all four waves to create a cross-sectional dataset of 21681 individuals. To minimize measurement errors related to the community education environment, we retain only communities with at least ten total households to ensure a reliable calculation of average community-level adult children’s education. Because our study specifically examines the effects of community education on older adults, we further restrict the sample to communities with at least three households that include respondents aged ≥60 years.

The data underwent a standardized cleaning process, followed by longitudinal merging across waves. For households with multiple children, we retained only the most educated child. If more than one child had the highest level of education, the eldest was selected, following evidence from Zimmer et al.^15^ and Ma^7^ that the highest educated and eldest child is most strongly associated with parental health outcomes. Given that the eldest child is more likely to have completed schooling and assumed family responsibilities, we focus on them as the primary influence on older adults’ behaviors, including tobacco use. After excluding samples with missing data, we merged individual, household, and provincial-level information, focusing on adult children aged 21–57 years.

**Figure 1:**
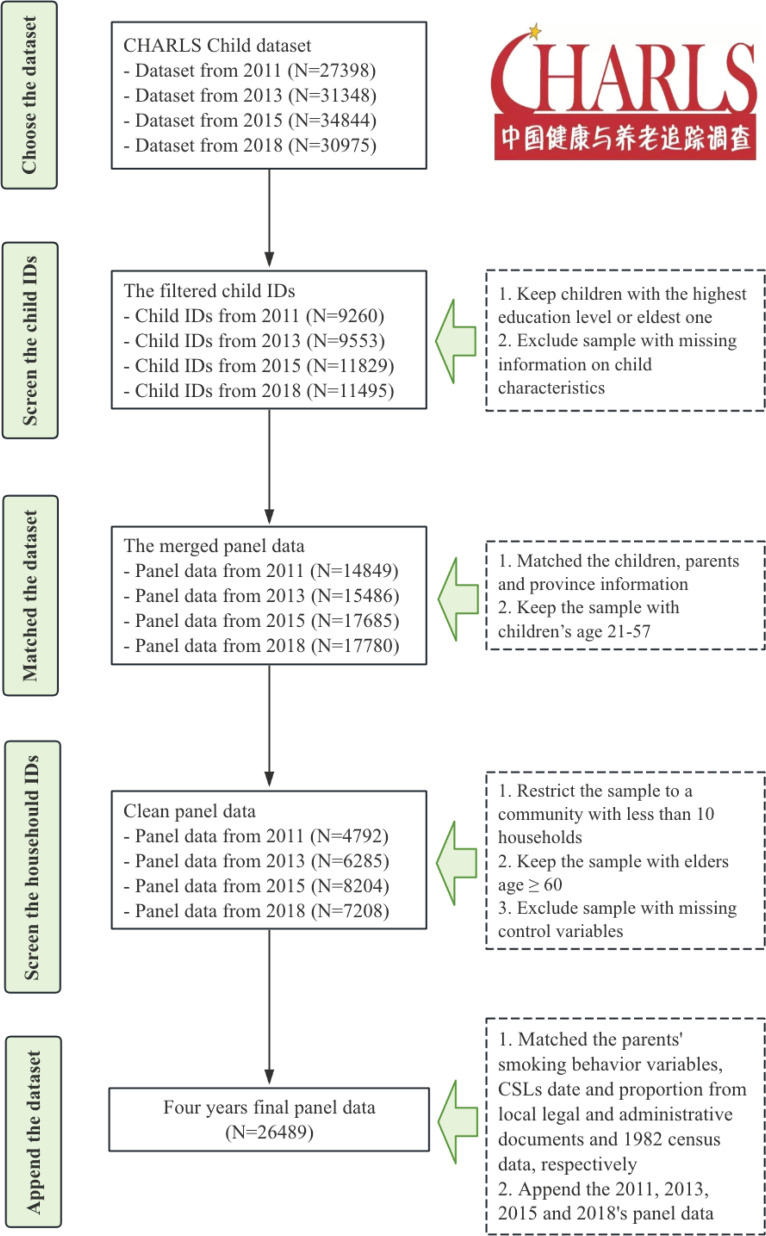
Analytical sample selection criteria, CHARLS, 2011–2018 (N=26489)

Following Ma^7^, Du et al.^16^, and Liang and Yu^17^, we generate an instrumental variable (IV) for community education based on the 1986 Compulsory Schooling Laws in China. These laws mandated nine years of compulsory education and were implemented gradually across provinces. The IV captures variations in the timing of implementation and the proportion of the population aged 16–18 years with fewer than nine years of education, based on the 1982 census. The final cleaned dataset was constructed by merging the four waves longitudinally, as illustrated in Figure 1.

### Measures

*Dependent variable*

Smoking behavior was measured using three variables. First, we assessed smoking history using the question: ‘Have you ever chewed tobacco, smoked a pipe, smoked self-rolled cigarettes, or smoked cigarettes/cigars?’. This variable is binary (0/1), indicating whether respondents have a history of any smoking behavior. Second, we evaluated current smoking status with the question: ‘Do you still have the habit or have you totally quit?’. This variable is also binary (0/1) and reflects whether respondents are currently smoking. Lastly, we quantified cigarette consumption by asking: ‘In one day, about how many cigarettes do/did you consume (now/before totally quitting)?’. This continuous variable provides a measure of the daily cigarette intake among current smokers.

*Explanatory variables*

This article examines the community education environment, with the primary explanatory variable defined as the average years of education among the adult sons and daughters of other older adults residing in the community (hereafter referred to as ‘community education’)^18^. The community serves as the fundamental unit of social interaction, where residents share common interests and a similar living environment. This proximity fosters daily social interactions and information exchanges, resulting in a distinct neighborhood effect.

*Covariates*

We controlled for a range of demographic characteristics of older adults that are strongly correlated with smoking status. These include gender, marital status, and education level measured in years of schooling. Additionally, we included the age of the child with the highest education level, along with the child’s birth year, gender, and marital status. At the community level, we controlled for the presence of a hospital within the community, the number of primary schools, and the population of individuals aged ≥65 years residing in the community.

### Statistical analysis

All statistical analyses were conducted using STATA version 18.0. Descriptive statistics were used to summarize smoking behaviors, community education level, and covariates across survey waves. Categorical variables are reported as frequencies and percentages, and continuous variables are presented as means with standard deviations. To identify the causal impact of community education on older adults’ smoking behavior, we employed an instrumental variable (IV) approach using two-stage least squares (2SLS) regression. The instrument was constructed based on the differential exposure to China’s CSLs across provinces and birth cohorts. All models included fixed effects for child birth year, survey year, and province-by-birth-year combinations, with robust standard errors clustered at the province-birth year level. The primary outcome variables include ever smoking, current smoking, and the number of cigarettes smoked per day.

Mediation effects were examined using both the Sobel test and structural equation modeling (SEM) to assess whether intergenerational support, well-being, and mental health mediate the relationship between community education and smoking outcomes. Standardized path coefficients and model fit indices were reported to evaluate SEM adequacy. We also explored heterogeneity by adding interaction terms and conducting subgroup analyses by province, based on the median exposure level to the CSLs reform. Additionally, 95% confidence intervals were included for all key coefficient estimates to improve result transparency. A two-sided p<0.05 was considered statistically significant. For the specific regression equation see Supplementary file Section A.

## RESULTS

### Descriptive results

The sample consists of adult children born between 1961 and 1989 (n=26489), identified as the highest educated children in their families, with parents aged ≥60 years at the time of the survey. The adult children in the sample are aged ≥22 years, ensuring they have completed their basic education and are likely to have living parents.

Table 1 presents the summary statistics of key variables. Among the respondents, 39.33% (n=8528) reported having smoked at some point, while 24.39% (n=4884) were identified as current smokers. The mean log of the number of cigarettes consumed per day among smokers is 0.86. The average community education level is 10.60 years, reflecting the educational environment in which these families reside. Additionally, 49.96% (n=13234) of respondents are female, and 82.37% (n=21820) are married. The education level of parents shows that 78.30% (n=20741) completed only elementary school or lower, whereas 25.67% (n=6800) of their children achieved a higher level of education. The correlation analysis of all variables is reported in Supplementary file Table 2.

**Table 1:** Descriptive statistics of major variables, CHARLS, 2011–2018 (N=26489)

*Variables*	*Sample*				
	*n*	*%*	*SE (%)*	*95% CI (%)*	
				*Lower*	*Upper*
**Smoking behavior**					
**Ever smoked**	21681	100			
Yes	8528	39.33	0.33	38.68	39.98
No	13153	60.67	0.33	60.02	61.32
**Current smoker**	20021	100			
Yes	4884	24.39	0.30	23.80	24.99
No	15137	75.61	0.30	75.01	76.20
	***n***	***Mean***	***SD***	***Min***	***Max***
Log of average cigarettes/day	21681	0.860	1.324	0	3.932
Number of cigarettes/day among current smokers	4884	13.98	11.12	0	50
**Independent variable and instrument variable**					
Community education	26489	10.60	1.918	0	18
Community (reform exposure × proportion)	26489	0.245	0.078	0.025	0.536
**Parent characteristics**					
**Gender**	26489	100			
Male	13255	50.04	0.31	49.44	50.64
Female	13234	49.96	0.31	49.36	50.56
**Marital status**	26489	100			
Married	21820	82.37	0.23	81.91	82.83
Widowed/divorced/separated	4669	17.63	0.23	17.17	18.09
**Years of schooling of parents**	26489	100			
Elementary school or lower	20741	78.30	0.25	77.80	78.80
Junior high school	3792	14.32	0.22	13.89	14.73
High school	1633	6.16	0.15	5.88	6.45
Junior college or higher	323	1.22	0.07	1.09	1.35
**Child characteristics**					
**Child birth year**	26487	100			
1955–1969	7783	29.38	0.28	28.83	29.93
1970–1979	12286	46.38	0.31	45.78	46.98
1980–1996	6420	24.24	0.26	23.72	24.75
**Gender**	26489	100			
Male	15625	58.99	0.30	58.39	59.58
Female	10864	41.01	0.30	40.42	41.61
**Marital status**	26489	100			
Married	23828	89.95	0.18	89.59	90.32
Widowed/divorced/separated	2661	10.05	0.18	9.68	10.41
**Years of schooling of the highest educated child**	26489	100			
Elementary school or lower	6800	25.67	0.27	25.14	26.20
Junior high school	8879	33.52	0.29	32.95	34.09
High school	5410	20.42	0.25	19.94	20.91
Junior college or higher	5400	20.39	0.25	19.90	20.87
**Community characteristics**					
Whether there is a hospital in the community	26489	100			
Yes	21682	81.85	0.24	81.39	82.32
No	4807	18.15	0.24	17.68	18.61
	***n***	***Mean***	***SD***	***Min***	***Max***
Number of primary schools	26489	0.707	0.825	0	6
Number of people aged ≥65 years in the community × 0.001	26489	0.396	0.492	0.018	5.60

SE: standard error. SD: standard deviation.

### Impact assessment based on instrumental variable method

We first validate the instrument by examining its relationship with community education. Supplementary file Figure 1 confirms a positive correlation between exposure to Compulsory Schooling Laws (CSLs) and community education level.

Table 2 reports the impact of community education on smoking behavior, measured across three outcomes: ever smoked, current smoker, and the log of average daily cigarette consumption. The ordinary least squares (OLS) model shows that higher community education level significantly decreases the likelihood of ever smoking (β= -0.014; 95% CI: -0.019–0.009). This effect is amplified in the two-stage least squares (2SLS) model, with the coefficient increasing (β= -0.030; 95% CI: -0.047 – -0.013).

**Table 2:** Effects of community education on smoke behaviors of older parents, CHARLS, 2011–2018

*Variables*	*Ever smoked* *(N=21681)*		*Current smoker* *(N=20021)*		*Log of average cigarettes per day* *(N=21681)*	
	*Coefficient (95% CI)*		*Coefficient (95% CI)*		*Coefficient (95% CI)*	
	*OLS*	*2SLS*	*OLS*	*2SLS*	*OLS*	*2SLS*
**Community education**	-0.014*** (-0.019 – -0.009)	-0.030*** (-0.047 – -0.013)	-0.012*** (-0.017 – -0.008)	-0.020** (-0.038 – -0.001)	-0.019*** (-0.031 – -0.007)	-0.038* (-0.081–0.006)
p	<0.001	0.001	<0.001	0.034	0.002	0.092
**Years of schooling of the highest educated child**	-0.005*** (-0.007 – -0.003)	-0.003** (-0.006 – -0.000)	-0.007*** (-0.009 – -0.005)	-0.006*** (-0.009 – -0.003)	-0.004 (-0.010–0.001)	-0.002 (-0.009–0.006)
p	<0.001	0.029	<0.001	<0.001	0.106	0.640
**Child’s gender**	0.010 (-0.005–0.024)	0.012* (-0.002–0.026)	-0.001 (-0.016–0.013)	-0.000 (-0.015–0.015)	0.038** (-0.000–0.076)	0.041** (0.003–0.079)
p	0.189	0.095	0.842	0.964	0.047	0.035
**Child’s marital status**	0.009 (-0.012–0.030)	0.008 (-0.013–0.029)	0.014 (-0.009–0.036)	0.013 (-0.009–0.036)	-0.000 (-0.057–0.056)	-0.002 (-0.058–0.055)
p	0.387	0.437	0.229	0.242	0.987	0.959
**Years of schooling of parents**	-0.000 (-0.003–0.002)	0.001 (-0.002–0.003)	-0.000 (-0.002–0.002)	0.000 (-0.002–0.003)	0.008*** (0.002–0.013)	0.010*** (0.003–0.016)
p	0.663	0.518	0.859	0.760	0.003	0.004
**Parents’ gender**	-0.518*** (-0.534 – -0.501)	-0.514*** (-0.531 – -0.497)	-0.358*** (-0.375 – -0.342)	-0.356*** (-0.373 – -0.339)	-1.269*** (-1.315 – -1.223)	-1.265*** (-1.312 – -1.217)
p	<0.001	<0.001	<0.001	<0.001	<0.001	<0.001
**Parents’ marital status**	-0.021** (-0.038 – -0.004)	-0.023*** (-0.040 – -0.006)	-0.030*** (-0.047 – -0.014)	-0.031*** (-0.048 – -0.015)	-0.038* (-0.082–0.006)	-0.040* (-0.084–0.003)
p	0.013	0.007	<0.001	<0.001	0.089	0.071
**Number of primary schools in the community**	-0.002 (-0.012–0.008)	-0.002 (-0.012–0.009)	-0.004 (-0.014–0.006)	-0.004 (-0.014–0.006)	-0.063*** (-0.088 – -0.038)	-0.063*** (-0.088 – -0.038)
p	0.724	0.736	0.479	0.465	<0.001	<0.001
**Whether there is** **a hospital in the community**	0.020* (-0.002–0.041)	0.025** (0.002–0.047)	0.026** (0.005–0.048)	0.029** (0.007–0.051)	0.070** (0.013–0.128)	0.076** (0.018–0.134)
p	0.070	0.031	0.016	0.011	0.017	0.010
**Number of people aged ≥65 years in the community**	0.001 (-0.016–0.018)	0.013 (-0.078–0.032)	0.012 (-0.050–0.029)	0.017 (-0.042–0.039)	0.039* (-0.053–0.008)	0.052* (-0.022–0.107)
p	0.871	0.226	0.168	0.114	0.085	0.060
**Birth province, survey year and child birth year FE**	Yes	Yes	Yes	Yes	Yes	Yes
R^2^	0.310	NA	0.213	NA	0.298	NA
**First-stage F-statistic**	NA	360.7	NA	328.5	NA	360.7

All regressions include individual controls described in Table 1, as well as a set of fixed effects. OLS: ordinary least squares model. 2SLS: two-stage least squares model.

* p<0.1.

** p<0.05.

*** p<0.01. NA: not applicable.

For current smoking status, the OLS estimate remains significant (β= -0.012; 95% CI: -0.017 – -0.008), and the 2SLS model shows a similar trend (β= -0.020; 95% CI: -0.038 – -0.001). Additionally, when examining the log of average cigarettes per day, the OLS estimate is -0.019 (95% CI: -0.031 – -0.007), while the 2SLS model indicates an even stronger effect (β= -0.038; 95% CI: -0.081–0.006).

The robustness of the 2SLS estimates is confirmed through the first-stage F-statistics, which indicate strong instruments, particularly for the years of schooling of the highest educated child (F-statistic=101.54). Overall, these results highlight the critical role of community education as a protective factor against tobacco use, suggesting that enhancing educational opportunities may lead to significant public health benefits by lowering both smoking prevalence and consumption levels.

### Robustness checks

We conduct a series of robustness checks to further validate the reliability of our findings and the plausibility of the exclusion restriction. First, we include four additional economic and healthcarerelated controls – GDP per capita, population growth rate, length of railways in operation, and number of doctors per 10000 people – in the main regression. The results remain stable, as reported in Supplementary file Table 3, suggesting that our estimates are not driven by omitted regional development factors. Second, we explore the heterogeneity of the IV’s effect by splitting provinces based on the median proportion of participants aged 16–18 years with less than nine years of schooling in 1982. Results in Supplementary file Table 4 show that the negative effects of community education on smoking are more pronounced in provinces with lower proportions. Third, we re-estimate the main specifications with province-by-survey-year fixed effects to account for time-varying regional shocks. As shown in Supplementary file Table 5, the results remain robust. Together, these checks provide further confidence in the validity of our empirical strategy.

### Mediation analysis

Sobel mediation analyses in Table 3 were conducted to investigate the mediating roles of three key variables: contacts of children (measured by the frequencies of other social contacts between parents and adult children via telephone calls, text messages, or mail/emails), optimism about future life, and score on the Center for Epidemiologic Studies Depression Scale (CES-D)^19,20^.

**Table 3:** Sobel test of mediation for intergenerational support, well-being, and mental health to smoke behaviors of older parents, CHARLS, 2011–2018 (N=26489)

*Mediating variables*	*Contacts of children*	*Optimism about future life*	*Mean of CES-D*
	*Coefficient (95% CI)*	*Coefficient (95% CI)*	*Coefficient (95% CI)*
**A: Ever smoked**			
Total effect	-0.0166*** (-0.020 – -0.013)	-0.0143*** (-0.018 – -0.011)	-0.0122*** (-0.016 – -0.008)
Direct effect	-0.0163*** (-0.020 – -0.012)	-0.0142*** (-0.018 – -0.010)	-0.0126*** (-0.016 – -0.009)
Indirect effect	-0.0003** (-0.0010 – -0.000)	-0.00001** (-0.0002 – -0.0000)	0.0004*** (0.0000–0.0010)
Proportion of total effect	0.018	0.007	-0.035
**B: Current smoker**			
Total effect	-0.0132** (-.017 – -0.009)	-0.0125*** (-0.016 – -0.009)	-0.0118*** (-0.016 – -0.008)
Direct effect	-0.0129*** (-0.017 – -0.009)	-0.0124*** (-0.016 – -0.009)	-0.0120*** (-0.016 – -0.008)
Indirect effect	-0.0003** (-0.0010–0.0000)	-0.0001** (-0.0002–0.0000)	0.0002* (-0.0000–0.0000)
Proportion of total effect	0.022	0.008	-0.014
**C: Log of average cigarettes per day**			
Total effect	-0.0182*** (-0.029 – -0.008)	-0.0192*** (-0.029 – -0.009)	-0.0202*** (-0.031–0.010)
Direct effect	-0.0188*** (-0.029 – -0.008)	-0.0190*** (-0.029 – -0.009)	-0.0211*** (-0.032 – -0.011)
Indirect effect	0.0005 (-0.0003–0.0010)	-0.0002 (-0.0004–0.0000)	0.0008*** (-0.0003–0.0010)
Proportion of total effect	-0.03	0.010	-0.04

All regressions include individual controls described in Table 1, as well as a set of fixed effects. CES-D: Center for Epidemiologic Studies Depression Scale.

* p<0.1.

** p<0.05.

*** p<0.01.

In Table 3, among individuals who have ever smoked (A), all three mediators show statistically significant indirect effects, although their magnitudes vary. CES-D demonstrates the strongest and most consistent mediation (β=0.0004; 95% CI: 0.0000 –0.0010), accounting for 3.5% of the total effect. Contact with children also exhibits a small but significant indirect pathway (β= -0.0003; 95% CI: -0.0010 – -0.0000), while optimism shows a slightly positive indirect effect (β= -0.0001; 95% CI: -0.0002 – -0.0000), representing 0.7% of the total effect. These results suggest that while all three factors contribute to smoking outcomes, most of their influence is driven by direct rather than mediated effects.

Among current smokers (B), the indirect effect of contact with children remains significant (β= -0.0003; 95% CI: -0.0010–0.0000), accounting for 2.2% of the total effect. Optimism and CES-D also serve as mediators to a less extent, with indirect effects of β= -0.0001 (95% CI: -0.0002–0.0000) and β=0.0002 (95% CI: -0.0000–0.0000), accounting for 0.8% and -1.4% of the total effects, respectively. For smoking intensity (C), CES-D is the only mediator with a statistically significant indirect effect (β=0.0008; 95% CI: -0.0003–0.0010), explaining approximately 4% of the total effect. In contrast, contact with children and optimism do not significantly mediate the relationship for this outcome. Together, these findings underscore the nuanced and modest roles that intergenerational support and mental health play in the pathway through which community education influences tobacco use among older adults.

**Figure 2:**
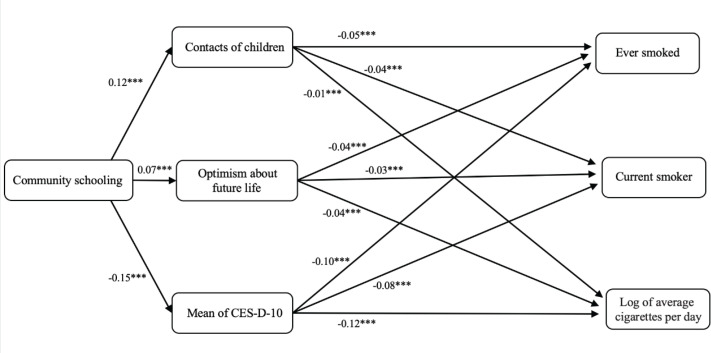
SEM, CHARLS, 2011–2018 (N=26489)

SEM is also used to test the mediating effects of intergenerational support, well-being, and mental health on the relationship between community education and older adults’ cognitive performance. The SEM results are presented in Figure 2 and include the standardized path coefficient estimates. The model demonstrates excellent fit, with fit indices of χ^2^=264.739 (df=96, p<0.001), CFI=0.951, TLI=0.912, RMSEA=0.015, GFI=0.922, AGFI=0.939, NFI=0.908, IFI=0.913, and PGFI=0.646. These indicators confirm that the model adequately fits the data and support the hypothesized relationships between community education, the mediators, and smoking behaviors among older adults.

### Moderation effects

Significant interactions are identified involving parents’ gender, the presence of a hospital in the community, the number of individuals aged ≥65 years, and number of primary schools in relation to smoking behaviors (Table 4). The results indicated that community education had a more substantial negative impact on smoking status among male respondents (for current smoker: β= -0.007; 95% CI: 0.000–0.014), and in communities with hospitals (for current smoker: β= -0.020; 95% CI: -0.029 – -0.012). Specifically, in communities with higher numbers of older adults and a greater number of primary schools, the negative effect of community education on current smoking status was pronounced (for ever smoked: β= -0.012; 95% CI: -0.021 – -0.003 and β= -0.004; 95% CI: -0.008–0.000; respectively).

**Table 4:** Interaction terms of community education and moderators, CHARLS, 2011–2018

*Variables*	*Ever smoked* *(N=21681)*				*Current smoker* *(N=20021)*				*Log of average cigarettes per day* *(N=21681)*			
	*Coefficient (95% CI)*				*Coefficient (95% CI)*				*Coefficient (95% CI)*			
**Community education**	-0.014*** (-0.019 – -0.009)	-0.016*** (-0.021 – -0.011)	-0.015*** (-0.020 – -0.010)	-0.025*** (-0.030 – -0.020)	-0.013*** (-0.018 – -0.008)	-0.014*** (-0.019 – -0.010)	-0.013*** (-0.018 – -0.008)	-0.020*** (-0.025 – -0.015)	-0.018*** (-0.030 – -0.006)	-0.020*** (-0.033 – -0.008)	-0.20*** (-0.032 – -0.000)	-0.038*** (-0.050 – -0.026)
p	<0.001	<0.001	<0.001	<0.001	<0.001	<0.001	<0.001	<0.001	0.004	0.001	0.002	<0.001
**Community education × Parents’ gender**	-0.006* (-0.013– 0.001)				-0.007** (0.000– 0.014)				-0.072*** (-0.091– -0.054)			
p	0.078				0.043				<0.001			
**Community education × Whether there is a hospital in the community**		-0.022*** (-0.031– -0.013)				-0.020*** (-0.029 – -0.012)				-0.013 (-0.036– 0.011)		
p		<0.001				<0.001				0.290		
**Community education × Number of people aged ≥65 years in the community**			-0.012*** (-0.021– -0.003)				-0.007 (-0.016– 0.001)				-0.021* (-0.041– 0.000)	
p			0.007				0.102				0.052	
**Community education × Number of primary schools**				-0.004* (-0.008– 0.000)				-0.008*** (-0.012 – -0.003)				-0.002 (-0.013– 0.009)
p				0.074				<0.001				0.738
**Fixed effects**	Yes	Yes	Yes	Yes	Yes	Yes	Yes	Yes	Yes	Yes	Yes	Yes
**Controls**	Yes	Yes	Yes	Yes	Yes	Yes	Yes	Yes	Yes	Yes	Yes	Yes
**R** **^2^**	0.312	0.313	0.313	0.072	0.216	0.217	0.216	0.062	0.303	0.300	0.300	0.101

All regressions include individual controls described in Table 1, as well as a set of fixed effects.

* p<0.1.

** p<0.05.

*** p<0.01.

## DISCUSSION

This study provides key insights into the relationship between community education and smoking behavior among older adults in China. The findings demonstrate that a higher level of education within a community is associated with lower smoking prevalence, supporting the notion that education serves as a protective factor against tobacco use. An additional year of community education, facilitated by exposure to CSLs, reduces the likelihood of ever smoking and current smoking by 3 and 2 percentage points, respectively, and decreases cigarette consumption by 3.8%. The IV estimate is nearly twice as large as the OLS estimate, which can be attributed to classical measurement error biasing OLS estimates toward zero, the IV capturing the local average treatment effect (LATE), and potential spillover effects 2022^7,16^. However, since both the key explanatory variable and the instrument vary at the community level, the influence of spillover effects is likely minimal in our analysis.

The observed impact of community education is particularly pronounced for current smoking behaviors and the amount smoked daily. These findings align with previous literature suggesting that educational attainment not only influences smoking initiation but also plays a role in reducing active smoking and cigarette consumption among users^21,22^. By highlighting the critical role of community education, this study suggests that investments in educational infrastructure could yield substantial public health benefits, potentially leading to lower smoking rates and associated health burdens^23^.

The mediation analysis highlights that the influence of community education level on smoking behaviors among older adults operates not only through direct pathways but also through modest yet meaningful indirect mechanisms. Specifically, intergenerational support – measured by contact with adult children – and mental health indicators such as depressive symptoms (CES-D) partially mediate the relationship, though their individual contributions are relatively small. CES-D consistently shows the strongest mediation effect, particularly for smoking intensity, suggesting that psychological well-being is a key channel through which education shapes health behavior in later life. These findings underscore the importance of addressing both social and emotional dimensions when designing community-level interventions aimed at reducing tobacco use among aging populations^24,25^.

**Figure 3:**
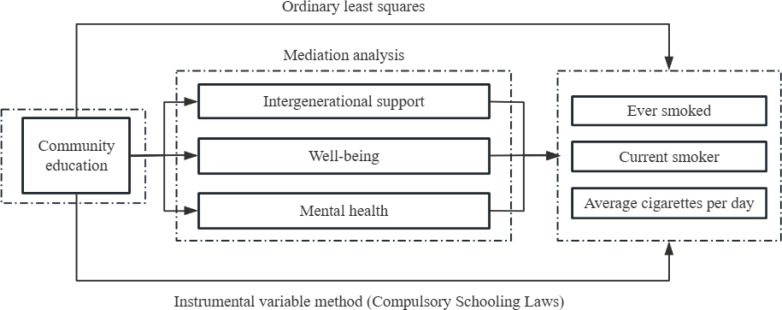
Theoretical framework

The moderation analysis reveals significant interactions involving parents’ gender and the community context, indicating that the effects of community education on smoking behavior vary across demographic groups. Specifically, the negative association between community education and smoking prevalence is stronger among male respondents and in communities with hospitals and more primary schools. This variation emphasizes the need for tailored interventions that consider the unique characteristics of different community settings and populations. The theoretical framework is shown in Figure 3.

These findings carry important policy implications. As countries work to combat smoking and its health impacts, strategies that promote education and improve access to healthcare services should be prioritized. Furthermore, integrating educational programs with health promotion initiatives could maximize their effectiveness in reducing tobacco use^26,27^.

### Limitations

This work has some limitations. First, there may be residual confounding due to unobserved individual- or community-level factors that simultaneously influence both community education level and smoking behaviors, such as individual self-control or broader socioeconomic conditions within the community. Second, although we cluster standard errors at the province-by-birth-year level to account for heterogeneity across regions and cohorts, potential spatial autocorrelation or clustering at the community level may remain unaddressed. This could lead to underestimated standard errors if unobserved factors are spatially correlated within communities. Third, the interpretation of our 2SLS estimates should be limited to the Local Average Treatment Effect (LATE). Since the instrumental variable – exposure to the Compulsory Schooling Laws – only affects a subset of individuals (i.e. compliers), the results may not generalize to the entire population. Therefore, caution is warranted in extrapolating these findings beyond the study sample.

## CONCLUSIONS

This study highlights the significant role of community education in reducing smoking behaviors among older adults in China. By employing instrumental variable methods, we demonstrate that higher levels of community education are associated with lower smoking prevalence and reduced cigarette consumption. These findings suggest that investing in education can serve as an effective public health strategy to combat tobacco use, particularly among men. As tobacco-related health issues remain a critical concern, our results advocate for policymakers to prioritize educational initiatives, as enhancing access to education could lead to substantial reductions in smoking rates and improved public health outcomes.

## Supplementary Material



## Data Availability

The data supporting this research are available from the authors on reasonable request.
