# Supplementary material for: The effects of community education on tobacco use among older adults in China
Source: Tob Induc Dis. 2025 May 29;23:10.18332/tid/204512. doi: 10.18332/tid/204512 (PMC12118464; doi:10.18332/tid/204512)
Supplement: Supplementary file 1 [file TID-23-71-s1.pdf]

Supplementary file

Section A Empirical model

To examine the intergenerational neighborhood effect of education, we first construct the following econometric model to estimate the intergenerational spillover effect of the average education level of other adult children at the community level on the smoking status of elderly individuals:

$$Y_{impt} = \beta_0 + \beta_1 \left(\frac{1}{N_m - 1}\right) \sum_{i \neq j} Cedu_{jmpt} + \beta_2 Cedu_{impt} + \beta_3 X_{impt} + \beta_4 Z_{mpt} + \theta_t + \tau_s + \tau_p \times \theta_t + \mu_{impt} \quad (1)$$

where Y_{impk} refers to the three smoking status indicators as described above for elderly parent i living in community m of province p whose highest-educated adult child was born in year t. $Cedu_{impt}$ is the years of schooling of the child of parent i. Our main variable of interest is $\left(\frac{1}{N_m - 1}\right) \sum_{i \neq j} Cedu_{jmpt}$, which is used to measure the educational environment of the neighborhood. The variable is the average years of education received by other highest-educated adult children in the community, which is calculated by dividing the total number of educational years by the total number of adult children in that community minus one (leave-out-mean). X_{impt} accounts for a number of child and parent characteristics that potentially influence parental outcomes, including parents' gender, marital status, and years of schooling, and children's birth year, gender, marital status, and years of schooling. Z_{mpt} accounts for community characteristics, including distance to the hospital dummy, number of primary schools, and number of older adults. θ_t are children's birth year fixed effects. τ_s are survey year fixed effects. $\tau_p \times \theta_t$ are province-birth year fixed effects. μ_{impt} is the error term, clustered at the province-birth year level.

Identifying this intergenerational neighborhood effect of education faces endogeneity problems, such as unobserved variables and self-selection. Following Du et al. (2021) and Liang and Yu (2022), we adopt the instrumental variable (IV) strategy by exploiting the exogenous variation induced by the CSLs implemented nationwide from 1986 to 1994.[16,17] Individuals who are more affected by CSLs are likely to present higher educational attainment. As different provinces implemented the reform at different times (Supplementary Table 1), we use adult children's exposure to compulsory education and (Exp_{impt}) its interaction with a measure of program intensity ($prop_p^{edu<9}$) as the instrument for children's education. Following Du et al. (2021),[16] we use a linear function for exposure to the CSLs to capture such a pattern and define Exp_{impt} as

$$Exp_{impt} = \begin{cases} 0, & if\ ChildCohort \leq FirstCohort_p; \\ \frac{ChildCohort-FirstCohort_p+1}{9}, & if\ FirstCohort_p \leq ChildCohor \leq FullCohort_p; \\ 1, & if\ ChildCohort > FullCohort_p \end{cases} \quad (2)$$

$prop_p^{edu<9}$ is measured as the proportion of the population aged 16-18 with less than 9 years of schooling in 1982.[18] Then, we construct the average number of CSLs reform to which the highest-educated adult child in the community (except for the focal adult's own children) was exposed, $\left(\frac{1}{N_m-1}\right)\sum_{i\neq j}(prop_p^{edu<9} \times Exp_{jmpt})$, as the IV for community education. The first-stage and second-stage regressions are as follows:

$$\left(\frac{1}{N_m-1}\right)\sum_{i\neq j} Cedu_{jmpt} = \gamma_0 + \gamma_1\left(\frac{1}{N_m-1}\right)\sum_{i\neq j}(prop_p^{edu<9} \times Exp_{jmpt}) + +\gamma_2 Cedu_{impt} + \gamma_3 X_{impt} + \gamma_4 Z_{mpt} + \theta_t + \tau_s + \tau_p \times \theta_t + \mu_{impt} \quad (3)$$

$$Y_{impt} = \delta_0 + \delta_1\left(\frac{1}{N_m-1}\right)\sum_{i\neq j} Cedu_{jmpt} + \delta_2 Cedu_{impt} + \delta_3 X_{impt} + \delta_4 Z_{mpt} + \theta_t + \tau_s + \tau_p \times \theta_t + \mu_{impt} \quad (4)$$

Supplementary Table 1. Implementation of Compulsory Schooling Laws by province

Province	Law effective year	First eligible birth cohort	Proportion of earlier cohort with less-9 years education
Beijing	1986	1971	0.066
Tianjin	1987	1972	0.14
Hebei	1986	1971	0.285
Shanxi	1986	1971	0.254
Inner Mongolia	1988	1973	0.318
Liaoning	1986	1971	0.224
Jilin	1987	1972	0.315
Heilongjiang	1986	1971	0.278
Shanghai	1987	1972	0.09
Jiangsu	1987	1972	0.284
Zhejiang	1986	1971	0.362
Anhui	1987	1972	0.477
Fujian	1989	1974	0.525
Jiangxi	1986	1971	0.488
Shandong	1987	1972	0.331
Henan	1987	1972	0.304
Hubei	1987	1972	0.341
Hunan	1991	1976	0.355
Guangdong	1987	1972	0.403
Guangxi	1991	1976	0.389
Chongqing	1986	1971	0.412
Sichuan	1986	1971	0.412
Guizhou	1988	1973	0.563
Yunnan	1987	1972	0.614
Shaanxi	1988	1973	0.328
Gansu	1991	1976	0.5
Qinghai	1988	1973	0.491
Xinjiang	1988	1973	0.354

The proportion of earlier cohort with less-9 years education is constructed based on 1% sample of 1982 census data.

Supplementary Table 2. Correlation matrix

Variables	1	2	3	4	5	6	7	8	9	10	11	12	13	14
Ever smoked	1													
Current smoker	0.786***	1												
Log of average cigarettes per day	0.559***	0.561***	1											
Community education	-0.067***	-0.059***	0.015**	1										
Community (reform exposure× proportion)	0.003	0.041***	0.023***	-0.092***	1									
Years of schooling of the highest educated child	-0.046***	-0.051***	0.024***	0.436***	-0.084***	1								
Child’s gender	0.015**	0.007	0.023***	0.034***	-0.017***	-0.078***	1							
Child’s marital status	-0.009	-0.018**	-0.023***	-0.016**	-0.026***	-0.049***	0.023***	1						
Years of schooling for parents	0.155***	0.130***	0.202***	0.333***	-0.123***	0.346***	0.053***	-0.057***	1					
Parents’ gender	-0.529***	-0.426***	-0.490***	0.005	-0.010	-0.026***	-0.005	0.025***	-0.347***	1				
Parents’ marital status	0.075***	0.057***	0.093***	0.027***	-0.042***	0.106***	0.019***	-0.008	0.145***	-0.182***	1			
Number of primary schools in the community	-0.002	0.007	-0.035***	-0.008	0.200***	0.006	-0.001	-0.012*	-0.007	0.002	-0.011*	1		
Whether there is a hospital in the community	0.007	0.015**	0.016**	0.091***	0.068***	0.029***	-0.003	0.015**	-0.013**	-0.005	0.014**	0.171***	1	
Number of people aged ≥65 in the community	-0.025***	-0.006	-0.003	0.256***	-0.068***	0.132***	0.030***	-0.022***	0.128***	0.008	0.013**	0.270***	0.125***	1

∗p < 0.1; ∗∗p < 0.05; ∗∗∗p < 0.01

Supplementary Table 3. Robustness Check of Macro variables (2SLS), CHARLS, 2011-2018

Variables (p-value)	Ever smoked (N=20,888)	Current smoker (N=19,266)	Log of average cigarettes per day (N=20,888)
	Coefficient (95% CI)	Coefficient (95% CI)	Coefficient (95% CI)
	(1)	(2)	(3)
Community education	-0.029*** (-0.046, -0.012)	-0.019** (-0.038, 0.001)	-0.039* (-0.083, 0.005)
	0.001	0.039	0.081
GDP per capita	-4.64*** (-5.87, 3.41)	-3.847*** (-5.08, -2.61)	-9.30*** (-12.9, -5.70)
	<0.001	<0.001	<0.001
Rate of population growth	0.366** (-0.044, 0.776	0.53** (0.085, 0.977)	-0.428 (-1.70, 0.850)
	0.080	0.019	0.512
Length of railways in operation (km)	16.4*** (12.1, 20.7)	13.7*** (9.34, 18.0)	33.0*** (20.35, 45.63)
	<0.001	<0.001	<0.001
Number of doctors (per 10000 persons)	0.974 (0.720, 1.23)	0.812*** (0.556, 1.06)	1.92*** (1.18, 2.67)
	<0.001	<0.001	<0.001
Fixed effects	Yes	Yes	Yes
Controls	Yes	Yes	Yes
First-stage F-statistic	355.5	323.3	355.5

Notes: All regressions include individual controls described in Table 1, as well as a set of fixed effects. Robust standard errors in parentheses are clustered at the province-birth year level.

$*p < 0.1$; $**p < 0.05$; $***p < 0.01$

Supplementary Table 4. Robustness Check of IV Heterogeneity by Province Subgroup Analysis (2SLS), CHARLS, 2011-2018

Variables (p-value)	Ever smoked (N=21,681)		Current smoker (N=20,021)		Log of average cigarettes per day (N=21,681)	
	Coefficient (95% CI)		Coefficient (95% CI)		Coefficient (95% CI)	
	Proportion ≥ median	Proportion < median	Proportion ≥ median	Proportion < median	Proportion ≥ median	Proportion < median
	(1)	(2)	(3)	(4)	(5)	(6)
Community education	-0.025** (-0.045, -0.004)	-0.063*** (-0.098, -0.028)	-0.012 (-0.035, -0.010)	-0.041** (-0.075, -0.006)	-0.031 (-0.083, -0.021)	-0.084* (-0.174, 0.005)
	0.018	<0.001	0.283	0.022	0.242	0.064
Fixed effects	Yes	Yes	Yes	Yes	Yes	Yes
Controls	Yes	Yes	Yes	Yes	Yes	Yes
First-stage F-statistic	236.9	103.1	215,2	89.03	236.9	103.1

Notes: All regressions include individual controls described in Table 1, as well as a set of fixed effects. Robust standard errors in parentheses are clustered at the province-birth year level.

$*p < 0.1$; $**p < 0.05$; $***p < 0.01$

Supplementary Table 5. Robustness Check of Add Province-Survey Year Fix Effect (2SLS), CHARLS, 2011-2018

Variables (p-value)	Ever smoked (N=21,681)	Current smoker (N=20,021)	Log of average cigarettes per day (N=21,681)
	Coefficient (95% CI)	Coefficient (95% CI)	Coefficient (95% CI)
	(1)	(2)	(3)
Community education	-0.031*** (-0.048, -0.014)	-0.016* (-0.035, 0.002)	-0.040* (-0.084, 0.004)
	<0.001	0.082	0.077
Birth province, survey year, province-survey year and child birth year FE	Yes	Yes	Yes
Controls	Yes	Yes	Yes
First-stage F-statistic	353.0	319.2	353.0

Notes: All regressions include individual controls described in Table 1, as well as a set of fixed effects. Robust standard errors in parentheses are clustered at the province-birth year level.

**p < 0.1; **p < 0.05; ***p < 0.01*

© 2025 Yu S. et al.
